# Treating Chronic Pain with SSRIs: What Do We Know?

**DOI:** 10.1155/2016/2020915

**Published:** 2016-07-03

**Authors:** Elias Patetsos, Emilia Horjales-Araujo

**Affiliations:** ^1^Copenhagen University, 2200 Copenhagen, Denmark; ^2^Department of Anesthesia, Center of Head and Orthopedics, Copenhagen University Hospital, 2200 Copenhagen, Denmark

## Abstract

Serotonin is a monoamine neurotransmitter that plays a major role in both nociception and mood regulation. Alterations in the 5-hydroxytryptophan (5HT) system have been reported in chronic pain patients. In recent years, Selective Serotonin Reuptake Inhibitors (SSRIs) have been suggested as an alternative treatment for chronic pain due to the fact that they are better tolerated presenting less secondary effects than other antidepressants such as tricyclic antidepressants. Although several clinical trials have been published, the effectiveness of SSRI as treatment for pain conditions is inconclusive. This review aims to summarise what is known, regarding the effectiveness of SSRI as a treatment for chronic pain conditions in adults. A total of 36 studies involving a total of 1898 participants were included in this review. Of the 36 trials included in the review, 2 used zimelidine as treatment, 3 used escitalopram, 4 used fluvoxamine, 4 used sertraline, 6 used citalopram, 8 used paroxetine, 9 used fluoxetine, and one used both citalopram and paroxetine. Because the trials included in this review are quite heterogeneous, only qualitative analyses were performed. SSRI seems to have an effect on most of chronic pain conditions; however, further clinical trials with good methodology leading to low risk of bias are needed in order to conclude once and for all the effect of this drug class as treatment for chronic pain conditions.

## 1. Introduction

According to the International Association for the Study of Pain, pain is defined as an unpleasant sensory and emotional experience associated with actual or potential tissue damage or described in terms of such damage [1]. Although there is no general consensus, chronic pain is accepted as pain that has lasted longer than three to six months [1]. Persistent or chronic pain seems to be reciprocally associated with depression and anxiety disorders; thus while chronic pain can lead to long lasting emotional disturbances, low mood state such as depression and anxiety increases the perception of acute and chronic pain [2–5].

Serotonin (5-hydroxytryptophan (5-HT)) is a monoamine neurotransmitter that plays a major role in both nociception and mood regulation [6–8]. Serotonin has long been associated with both central and peripheral regulation of the nociceptive signal [8, 9] and alterations in the 5HT system have been reported in chronic pain patients (for review see [10]). In recent years, considerable research efforts have focused on the role played by 5-HT and its respective receptors in processing and modulating noxious information [6–8]. The 5-HT system represents a powerful system that can both decrease and increase the magnitude of pain following noxious stimulation.

An important modulator of 5HT transmission is the serotonin transporter (5HTT), which is essential for determining the intensity and duration of the serotoninergic signal [11, 12]. Polymorphism in the serotonin transporter gene has been associated with altered pain experience [13, 14]. Antidepressants affecting the monoaminergic system are now part of the therapeutic strategy for treatment of several chronic pain conditions (for review see [15]). Selective Serotonin Reuptake Inhibitor (SSRI) is a family of antidepressants that exerts its action by inhibiting the reuptake of serotonin into the presynaptic neuron after serotonin has been released, affecting the duration and intensity of the serotonin communication [11]. In recent years, SSRIs have been proposed as alternative treatment for chronic pain due to the fact that they are better tolerated and present less secondary effects than other antidepressants such as tricyclic antidepressants (TCAs) [15]. Although several clinical trials have been published, the effectiveness of SSRIs as treatment for pain conditions is inconclusive. This review aims to summarise what is known, so far, regarding the effectiveness of SSRIs as a treatment for chronic pain.

## 2. Materials and Methods

**Figure 1:**
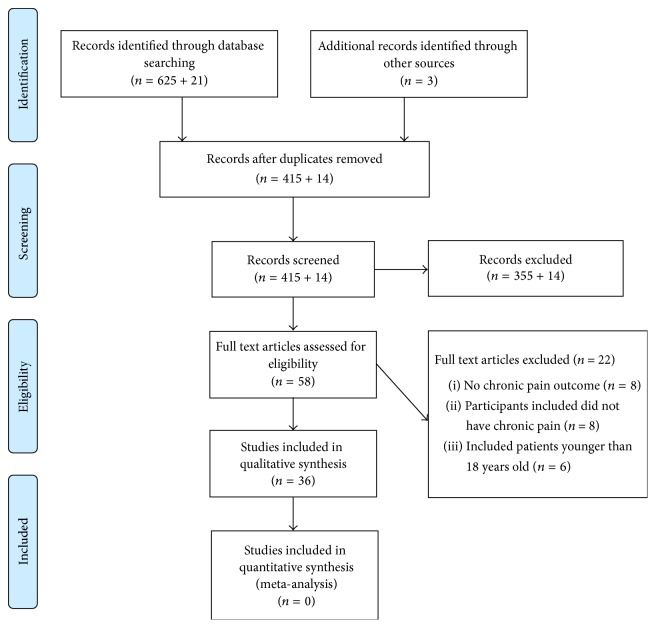
Diagram of the publications screening process based on PRISMA Statement (original search + search 6 months after).

A detailed description of the methods is published in the PROSPERO database under registration number CRD42014013777. In summary, studies that appeared potentially relevant were identified by literature search in the PubMed and Cochrane databases by the terms presented in supplementary Table 1 in Supplementary Material available online at http://dx.doi.org/10.1155/2016/2020915. Following Cochrane suggestions, a second search of published studies was carried out 6 and 12 months after the initial search (June 2015 and January 2015, resp.). A flow diagram of the screening process based on PRISMA Statement is presented in Figure 1. Studies were included for revision if they were clinical trials analyzing the effectiveness of SSRIs as treatment for chronic pain conditions in adult patients (intervention group). Patients receiving any placebo (containing no active substance) or any active substance employed to ameliorate pain outcome as well as patients not receiving a treatment were used as control group. Studies were excluded from the review if they were not clinical trial articles published in English, if they did not include chronic pain patients, or if they did not have pain assessment (e.g., pain intensity or analgesic consumption) as outcome. No authors were contacted for further data, and no study protocols or original data were examined.

The results of the literature search were evaluated firstly by screening the study titles and, subsequently, by screening the abstracts of the possible eligible studies. After the abstract screening, full text screening of possible eligible studies was performed. Data was extracted into an excel datasheet in order to minimise subjectivity. Extracted data included study design (presence of placebo arm, blinding, randomisation, and cross-over), number of patients, chronic pain conditions experienced by the patients, SSRI used as treatment, duration of the trial, documented adverse effects and change in pain score outcomes (e.g., intensity, frequency, and analgesic consumption), and primary outcome (Tables 1(a) and 1(b)). All aspects of the literature review process (e.g., screening, data extraction, and quality assessment) were carried out by two independent investigators. Quality of the included studies and presence of bias were assessed based on five domains proposed by the Cochrane Collaboration tool for assessing risk of bias (random sequence generation, allocation concealment, blinding of participant and personnel and of outcome assessment, incomplete outcome data, and selective reporting).

## 3. Results

A total of 58 studies were considered, of which 36 met the inclusion criteria. Of the 22 excluded studies, 6 included nonadults patients, 8 studies included patients with acute or experimental pain (nonchronic pain patients), and 8 subjects did not measure pain outcome. A total of 36 studies involving a total of 1898 participants were included in this review. The distribution of the 1898 patients included in pain conditions were as follows: 259 patients with fibromyalgia, 166 with somatoform pain disorder, 280 with chronic low back pain, 467 with chronic tension type headache or migraine, 103 with chronic pelvic pain, 42 with prostatodynia, 195 with noncardiac chest pain, 204 with diabetic painful neuropathy, 48 with painful polyneuropathy, 31 with central poststroke pain, and 40 with chronic musculoskeletal pain, and 63 participants included in two studies were not classified into type of chronic pain condition. The total number of patients included in the studies varied from 14 to 122 (see Table 2). Of the 36 trials included in the review, 2 used zimelidine as treatment, 3 used escitalopram, 4 used fluvoxamine, 4 used sertraline, 6 used citalopram, 8 used paroxetine, 9 used fluoxetine, and one used both citalopram and paroxetine. Because the trials included in this review are quite heterogeneous, only qualitative analyses were performed.

### 3.1. Risk of Bias

While nine of the trials have one “unclear” risk of bias, 23 trials presented one or more domain at “high risk” or at least two domains with “unclear” risk; and only four trials were evaluated to have “low risk” in all domains (see Table 3). Only two of the four trials with “low risk” of bias reported a significant effect of SSRI as treatment for chronic pain and fourteen of the studies at “high risk” of bias reported a significant effect of SSRI.

### 3.2. Effect of SSRI as Treatment for Chronic Pain

As shown in Table 2, six studies presented contradictory or inconclusive data (e.g., a reduction of analgesic consumptions but not on pain intensity was observed; effect on pain symptoms was observed by the physician but not on self-reported pain intensities). Five studies found no effect of the SSRI on pain outcomes. Two of these studies were done in larger samples of patients [16, 17] and only one mentioned sample size and power calculations [17]. The other four studies were done in samples of less than 40 patients and did not mention any sample size calculation; it is thus possible that these studies might be underpowered. Finally, the other 26 studies found a significant effect of the SSRI on chronic pain outcomes. Interestingly, all five studies analyzing the effect of fluvoxamine described a significant effect of the SSRI on pain outcomes. Similarly, all three studies using escitalopram reported a significant positive outcome.

To date, fluoxetine is the most studied SSRI in relation to chronic pain treatment. Although there are no studies reporting an insignificant effect of this SSRI, two trials found contradictory results, reporting that fluoxetine either had similar effect as desipramine (TCA) on chronic tension type headache [18] or had an effect of the SSRI on overall headache but not on migraine [19].

### 3.3. Zimelidine

Zimelidine was the first SSRI antidepressant to be produced. Although the drug had very significant effects as antidepressant, within a year and a half of its introduction, some strong secondary effects (e.g., Guillain-Barré syndrome) were reported to be associated with the drug, forcing the withdrawal of the drug from the market [20]. There were, however, two studies that analysed the effect of zimelidine on chronic pain outcomes. Of those two studies, one observed a significant effect of the SSRI on pain relief (measured as pain intensity) and reduction in analgesic consumption compared with placebo [21]. However, the other study reported inconclusive results, while zimelidine significantly reduced pain outcome assessed by the physician; there were no significant differences in self-rated pain by the patients while consuming the drug compared to placebo, VAS 45.7 ± 24.6 and 45.0 ± 27.0, respectively [22].

### 3.4. Sertraline

Sertraline has mainly been used to treat depression and obsessive-compulsive disorders. Although sertraline is associated with a higher rate of side effects [23–25], it has comparatively lower risk of drug interactions and can be combined with analgesics. Four trials analysed the possible effect of sertraline as chronic pain treatment. Two studies reported a significant effect of sertraline in noncardiac chest pain, measured in pain intensity and unpleasantness [26, 27]. These two studies showed no significant change in mood between sertraline and a placebo group, suggesting that the effect of the SSRI on pain outcomes is not associated with an improvement in mood. On the offside, the rate of side effect reported in these two trials was quite high. A third study found an effect of sertraline on pain outcomes in males with chronic pelvic pain syndrome compared to baseline, but the difference was not significant when the intervention group was compared to placebo [28]. Finally, a fourth study reported that although sertraline slightly significantly improved the emotional state of the patients, the treatment had no statistically significant effect on the pain outcomes (pelvic pain intensity) [29].

### 3.5. Citalopram

Citalopram has been described to have antidepressant properties similar to tricyclic drugs but with significantly less side effects [30]. In animal models citalopram has been associated with analgesic effects [31]. However, in humans controversial data has been observed. Seven studies analysed the effect of citalopram on chronic pain outcomes. Two of the seven studies found a significant effect of the SSRI as treatment for chronic pain: somatoform pain disorder [32] and diabetic neuropathy [33], measured as pain intensity (VAS), total pain rating index, and observed and self-rated pain intensity and symptoms, respectively. Three studies found no effect in patients with chronic tension type headache [34], chronic pelvic pain [35], and fibromyalgia [36], measured as area under the headache curve, pain disability index, and McGill pain questionnaire and pain tender points and fibromyalgia symptoms, respectively. In addition, two studies reported inconclusive results on self-rated pain in patients suffering from painful diabetic neuropathy [37] and fibromyalgia [38].

### 3.6. Fluoxetine

Fluoxetine was the third most prescribed antidepressant after sertraline and citalopram in 2010 [39]. Its effect on serotonin system and receptors is well known but its effect on other receptors is not well understood. In an open-labelled, placebo-controlled trial, Gordon and colleagues found that fluoxetine given 7 days before molar surgery inhibited the analgesic effect of morphine; therefore the authors suggested an SSRI action on the mu (*μ*) receptors [40].

Ten studies included in this review used fluoxetine as treatment for different chronic pain conditions. Nine trials reported a positive effect of the SSRI on chronic pain outcomes: fibromyalgia [41, 42] (measured by pain scores and Fibromyalgia Impact Questionnaire scores), chronic tension type headache [18] patients (measured as self-reported improvement), migraine without aura [43] (total pain index), persistent somatoform pain disorder [44] (medical outcomes study pain scores), painful diabetic neuropathy [45] (measured as self-rated pain), musculoskeletal pain [46] (measured as pain intensity and relief), chronic pelvic pain syndrome [47] (chronic prostatitis index), and chronic daily headache [19] (measured as VAS pain intensity). An additional study reported inconclusive results on migraine [19] (measured as VAS pain intensity).

### 3.7. Paroxetine

When released, paroxetine was the most potent and selective of all SSRI available [48]. A total of nine studies examined the use of paroxetine as treatment for chronic pain. Four studies found an amelioration of pain outcomes: self-rated pain intensity by noncardiac chest pain patients [49], chronic headache [50, 51], fibromyalgia [52] measured by Fibromyalgia Impact Questionnaire, and pain intensity experienced by diabetic neuropathy patients [53]. On the other hand, two studies found no effect of the SSRI on pain intensity in chronic low back patients [16, 17] and three trials described inconclusive results in chronic tension type headache [54] (measured by days where the patients experienced headache), chronic headache [50, 51], and self-reported pain improvement by painful diabetic neuropathy patients [37].

### 3.8. Fluvoxamine

Fluvoxamine is a potent and selective SSRI with approximately 100-fold affinity for the serotonin transporter over the norepinephrine transporter [55]. Three publications were found to analyse the effect of fluvoxamine on chronic pain outcomes. All three studies found a positive effect of the drug on chronic tension type headache [56] (assessed by frequency of headaches and pain severity), self-rated pain duration and intensity by prostatodynia patients [57], and central poststroke pain [58] measured by pain intensity.

### 3.9. Escitalopram

Escitalopram is the (S)-stereoisomer (enantiomer) of citalopram, hence the name. Some studies suggest that escitalopram might be more effective than citalopram in treating depressed patients [59, 60]. All three studies analysing the effect of escitalopram as treatment for chronic pain reported positive results: chronic lower back pain [61] measured as weekly pain relief, multisomatoform disorder (pain intensity) [62] and painful polyneuropathy [63] (evaluated by self-rated pain relief).

## 4. Discussion

Serotonin (5HT) is a monoamine neurotransmitter that plays a major role in both nociception and mood regulation [6–8]. A major player in 5-HT signalling is the serotonin transporter (5-HTT), which is essential for determining the 5-HT level at the postsynaptic receptor (for review see [11]). SSRIs act upon the 5-HTT inhibiting the reuptake of the monoamine into the presynaptic cell, increasing the level of serotonin in the synaptic cleft. In the past decades, SSRIs have emerged as alternative treatment for chronic pain but their effectiveness is inconclusive.

This topical review aimed to summarise what is known about the effectiveness of the use of SSRI as treatment for chronic pain. A total of 36 trials were included in this revision. Twenty-five studies reported a significant effect of SSRI on chronic pain outcomes. However, only two of these studied were categorised as having “low risk” of bias.

In general, most studies do not seem to be congruent on the methodology adopted and present a “high risk” of bias: lacking a control-group (placebo or other drugs), not including sample size calculation, lacking randomisation (or not describing method of randomisation), or even lacking blinding of the researcher and/or patients. To date, fluoxetine is the most studied SSRI in relation with chronic pain treatment. No studies reported an insignificant effect of this SSRI; however one study found contradictory results reporting an effect on overall headache but not on migraine [19].

More than 70% of the studies included in this review found a statistically significant effect of SSRI as treatment for chronic pain conditions. Fluoxetine, fluvoxamine, and escitalopram in particular seem to be the most promising SSRI. Further studies using those SSRIs in different concentrations and with a systematic methodology minimising bias (proper blinding, randomisation of patients, placebo (or active comparator) control, and perhaps a cross-over period) are of high importance in order to assure the use of optimal dosage and treatment periods in the clinical practice. Furthermore, the studies reviewed here were of short duration, varying from 2 to 36 weeks. Since chronic pain conditions often evolve into permanent pain (lasting for even a lifetime), future studies with longer treatment period are strongly encouraged.

Chronic pain and depression are highly prevalent conditions whose symptoms overlap. A large number of studies have found a reciprocal association between emotions (especially depression) and pain [64–69]. Since SSRIs are designed and used to treat depression and other psychological disorders, it would be of great interest to investigate if the effectiveness of SSRIs as treatment for chronic pain is mediated by its effect as mood regulator. Future studies focusing on how does SSRI effectiveness as pain treatment compare to that of TCAs and to the newer SNRIs are also needed. Perhaps SSRIs differ from TCAs as treatment for chronic pain conditions by having different effect on mood modulation. If SSRIs act on pain conditions by modulating the emotional state, longer treatment periods might be required in order to observe a positive effect. If this is the case, studies with short SSRI treatment duration (as many of the trials included in this review) would not be able to show a significant effect on the pain conditions. This strengthens the necessity of longer trials studying the effect of SSRI on mood states and chronic pain conditions.

Statistical significant effect of SSRI on chronic pain was not always observed; however the clinical significance of the relation between SSRI and chronic pain cannot be ruled out. The clinical significance of the SSRI effectiveness as treatment for chronic pain was not always analysed in the trials included in this revision. A statistical significance numerical difference in pain intensity will not always imply a clinical significance (patient's life quality). For example, one of the studies found a significant effect when pain outcome was measured by the physician but no statistical significance was seen when self-rated pain by the patient was analysed [22]. In this aspect it is also important to remark the trial analysing the effect of sertraline on pelvic pain intensity; while an improved emotional state was observed on the patients, the treatment had no statistically significant effect on the pain outcomes [29]. Thus, although the patients' life quality improved by improving their mood, the pain intensity reminded unchanged. This opens to the discussion of whether a statistically significant physiological effect of SSRI on pain outcomes is enough to generate a clinical significant effect (e.g., emotional state) or not, and vice versa.

Generally chronic pain patients have tried several treatments, feeling desolated and without hope of being pain-free. The fact that SSRIs have a meaningful improvement in pain symptoms for many patients involved in the trials, in addition to the SSRI's safety profile with low frequencies of adverse events, might open for the discussion of choosing SSRI over other drugs, for example, TCA or gabapentin, to treat chronic pain conditions in the clinical practice. Clinicians are recommended to analyse in a case by case basis whether the use of SSRI to treat chronic pain conditions might improve the patient's life quality, for example, in patients who have tried other treatments without success or patients who have been successfully treated with TCAs but had to discontinue the treatment due to the experienced adverse events. Patients with mild chronic pain conditions might also be beneficiated of having SSRI as first treatment due to the safety profile, before trying a more aggressive treatment as, for example, TCAs.

The present review has some limitations: first, the methodology adopted might have excluded data since only published articles in English were included; furthermore and in contradiction with PRISMA recommendations, unpublished clinical trials were not included in the present review; therefore potential studies may not have been included in this revision. Secondly, the authors were not contacted for additional data and clarification; perhaps by contacting the authors, more information could be gathered regarding the methodology used in each study, facilitating further analysis and conclusion. In addition, studies analysed in this review comprehended seven different SSRIs and a variety of chronic pain conditions and pain outcome measurements increasing the heterogeneity of the studied population. Finally, meta-analysis was not done, and only qualitative data is presented in this review.

A meta-analysis would be of great help in order to quantitatively measure the effect of SSRI on chronic pain conditions. However, precautions should be taken when performing the recommended meta-analysis. The high heterogeneity in the existing clinical trials in regard to the pain conditions, the pain outcome measurements, and the demographic of the patients studied, in addition to the poor risk of bias contingency, might lead to a poor statistical analysis. It is thus a priority to improve the quality and consistency of future clinical trials studying the effect of SSRI on chronic pain conditions including a control group (e.g., placebo, TCA, or gabapentin).

## 5. Conclusion

SSRI seems to have an effect on most of chronic pain conditions; however further clinical trials with a good methodology leading to low risk of bias are needed in order to conclude once and for all the effect of this drug as treatment for chronic pain conditions. In addition, it will be of great interest to continue this review with a meta-analysis study following PRISMA and Cochrane Collaboration guidelines in order to statistically asses the published data.

## Supplementary Material

Supplementary Table 1 presents the detailed search terms and results from the literature search. Filter restriction used was: clinical trials and English as language.

## Figures and Tables

**(a):** 

Reference	Blinded	Randomized	Cross-over	Placebo	Pain condition	Number of patients	SSRI	Total trial duration (in weeks)	Measured pain outcome	Reported results
[38]	Double	Yes	No	Yes	Fibromyalgia	40	Citalopram	20	Pain perception and Fibromyalgia Impact Questionnaire (FIQ)	After two months with citalopram treatment there was a significant decrease on pain outcomes (*P* < 0.05). After four months of treatment the effect diminished (being nonsignificant).

[41]	Double	Yes	No	Yes	Fibromyalgia women	60	Fluoxetine	12	Fibromyalgia Impact Questionnaire and pain intensity score	Pain intensity mean change from baseline to endpoint was −8.6 ± 14.5 for fluoxetine group and 2.9 ± 13.6 for placebo (*P* = 0.005).

[16]	Double	Yes	No	Yes	Chronic lower back pain	103	Paroxetine	8	Pain intensity	There was a 45% decrease in pain intensity on maprotiline, compared to 27% decrease in placebo, and 26% decrease on paroxetine. The mean reduction in pain intensity on paroxetine compared to placebo was not significant (*P* = 0.64).

[34]	Double	Yes	Yes	Yes	Chronic tension type headache	40	Citalopram	24	Intensity and duration of headache	During placebo, headache outcome decreased by 10% compared with baseline (*P* = 0.12). Pain outcome was 30% lower during amitriptyline when compared to placebo (*P* = 0.002) and 20% when compared to citalopram (*P* = 0.12 after Bonferroni correction).

[49]	Double	Yes	No	Yes	Noncardiac chest pain	50	Paroxetine	8	Pain intensity rating	Paroxetine treated patients showed greater improvements than placebo (*P* < 0.05) on the Clinical Global Impressions scale.

[29]	Double	Yes	Yes	Yes	Women with chronic pelvic syndrome	33	Sertraline	12	Pain intensity	Composite pain intensity score after sertraline 2.7; pain intensity after placebo 2.7^#^.

[43]	Double	Yes	No	Yes	Migraine without aura	52	Fluoxetine	28	Total pain index (TPI)	TPI was significantly reduced after fluoxetine treatment (41.3 ± 63.8) compared to start point (135 ± 115.8; *P* = 0.014).

[17]	Double	Yes	No	Yes	Low back pain	92	Paroxetine	8	Pain intensity	No significant effect on pain intensity between paroxetine (57 ± 23.8) and placebo (57 ± 24.3).

[22]	Double	Yes	Yes	Yes	Chronic pain (variety)	21	Zimelidine	12	Self-rated pain intensity and doctor's pain global assessment	Self-rated pain intensity after 6 weeks of zimelidine treatment 45.7 ± 24.6 and after placebo treatment 45.0 ± 27.0. There was a statistical significant difference in global assessment between zimelidine and placebo phases on the doctor's assessment (rate varies between pain conditions).

[42]	Double	Yes	Yes	Yes	Fibromyalgia	31	Fluoxetine	20	Pain intensity and physician evaluation in tender points	Pain intensity at baseline was 68.4 ± 20.4. Pain intensity was significantly decreased after fluoxetine treatment (47.6 ± 19.8) compared to placebo (58.8 ± 17.1; *P* < 0.001).

[21]	Double		No	Yes	Chronic pain (variety)	40	Zimelidine		Pain relief and analgesic consumption	Patients' self-rated pain decreased from 64.9 ± 6.3 at start to 46.8 ± 5.1 after zimelidine treatment (*P* < 0.01). Patients receiving placebo reported a start pain of 44.8 ± 5.4 and a final pain intensity of 46.6 ± 7.8 (nonsignificant change). The physician's clinical judgment of changes in level of pain showed that 12 patients were considered improved, 9 in zimelidine and 3 in the placebo group (*P* < 0.05).

[26]	Double	Yes	No	Yes	Noncardiac chest pain	115	Sertraline	34	Pain intensity and unpleasantness	The authors did not mention the raw pain data. However, they analyzed the results in terms of treatment condition × time interaction. The overall analysis was significant for both pain intensity [*F*(3,941) = 6.51, *P* < 0.001] and unpleasantness [*F*(8,870) = 6.21, *P* < 0.001]. Groups with coping skill training (CST) + sertraline or sertraline alone resulted in greater reductions in pain intensity and unpleasantness compared to placebo alone.

[45]	Double	Yes	Yes	Yes	Painful diabetic neuropathy	57	Fluoxetine	13	Self-rated pain relief	The mean pain-diary scores decreased by 0.35 ± 0.11 units in the patients consuming fluoxetine and 0.15 ± 0.07 units in patients receiving placebo (*P* < 0.05).

[50]	Double	Yes	Yes	No	Chronic tension type headache	87	Paroxetine	16	Headache intensity and analgesic consumption	No statistical significance between the effect of paroxetine and sulpiride. Paroxetine improved headache intensity (change in headache −0.4, *P* < 0.001) and analgesic consumption (change −0.8, *P* < 0.05) when compared to baseline.

[28]	Double	Yes	Yes	Yes	Males with chronic pelvic pain syndrome	14	Sertraline	26	Prostatic symptom severity (PSS) and prostatic symptom frequency (PSF)	PSS at baseline 23.4 and PSS after 13 weeks of sertraline 17.3 (*P* = 0.34). PSF at baseline 15.9 and PSF after sertraline treatment 12.3 (*P* = 0.09). No significance between sertraline and placebo treatment (PSF *P* = 0.41 and PSS *P* = 0.44)^#^.

[44]	Double	Yes	No	Yes	Persistent somatoform pain disorder	80	Fluoxetine	8	Medical Outcomes Study Pain Measures (MOSPM)	MOSPM total score after fluoxetine treatment (33.08 ± 18.81) was significantly reduced in comparison with baseline (59.53 ± 22.76; *P* < 0.01). Participants receiving fluoxetine had greater reduction in MOSPM total score when compared to placebo (MOSPM 65.75 ± 24.87 at baseline and 55.33 ± 25.44 at endpoint).

[62]	Double	Yes	No	Yes	Multisomatoform disorder	51	Escitalopram	12	Patient Health Questionnaire-15 score (PHQ), pain intensity (VAS)	There was a significant improvement in PHQ in both escitalopram (from 14.6 ± 0.96 to 5.6 ± 1.0, *P* < 0.05) and placebo (17.3 ± 0.9 to 12.5 ± 1.0, *P* < 0.05) at the end of the trial compared to baseline. There was also a significant difference between placebo (12.5 ± 1.0) and escitalopram group (5.6 ± 1.0, *P* < 0.05) at the end of the trial.

[36]	Double	Yes	No	Yes	Fibromyalgia	42	Citalopram	8	Pain intensity and Fibromyalgia Impact Questionnaire (FIQ)	Pain self-assessment for citalopram group at start was 6.3 ± 2 (change −1 ± 2.1) and for placebo group was 6.7 ± 1.9 (change −0,7 ± 1.1). No significant effects were observed between the two groups.

[63]	Double	Yes	Yes	Yes	Painful polyneuropathy	48	Escitalopram	10	Self-rated pain relief	Pain relief after 5 weeks of treatment with escitalopram was higher than during placebo, with a mean of 0.8 (*P* = 0.001).

[52]	Double	Yes	No	Yes	Fibromyalgia	86	Paroxetine	12	Fibromyalgia Impact Questionnaire (FIQ) total score	Significantly greater proportion of subjects in the drug group responded (56.8%) than in the placebo group (32.7%) regarding reduction in FIQ score (*P* = 0.016).

[57]	Double	Yes	No	Yes	Chronic prostatodynia	42	Fluvoxamine	8	Pain intensity	The authors did not report the improvements in pain in terms of percentage from baseline. The fluvoxamine-treated group showed significant improvement in pain when compared to placebo group (rank sum 553 at week 8, *U* = 322, *P* = 0.01). This significance was observed from week 4 (rank sum 528.5; *U* = 297.5, *P* = 0.05).

[19]	Double	Yes	No	Yes	Chronic daily headache and migraine	122	Fluoxetine	4 (single blind) + 12 (double blind)	Overall headache intensity and frequency	At the end of the trial the fluoxetine group showed a significant effect in headache improvements (*P* = 0.001) compared to placebo. No pain intensity in detail was published.

[53]	Double	Yes	Yes	Yes	Diabetic neuropathy	29	Paroxetine	6	Pain intensity	Pain intensity during placebo 5.79 and in fluvoxamine 1.25 (*P* = 0.01)^#^.

[27]	Double	Yes	No	Yes	Noncardiac chest pain	30	Sertraline	9	Pain intensity	Group 1 initial pain score 3.94; pain score after sertraline treatment 1.47 (*P* = 0.02). Group 2 initial pain score 3.50; pain score after placebo 2.96 (*P* = 0.58); significance difference between placebo and sertraline group (*P* = 0.02)^#^.

**(b):** 

Reference	Blinded	Randomized	Cross-over	Placebo	Pain condition	Number of patients	SSRI	Total trial duration (in weeks)	Measured pain outcome	Reported results
[32]	Double	Yes	No	No	Somatoform pain disorder	35	Citalopram	8	Self-assessed McGill pain questionnaire	In the citalopram group pain scores decreased significantly during the 8-week trial (41.9 ± 17.7 vs 90.0 ± 19.02, *P* = 0.004).

[35]	No	No	No	Yes	Women chronic pelvic pain	14	Citalopram	12	McGill pain intensity scale and pain disability index (PDI)	Pain severity showed a nonsignificant trend toward improvement on the McGill pain intensity scale (*P* = 0.096). There were no significant differences on the PDI (*P* = 0.158).

[51]	No	No	No	No	Chronic daily headache	60	Paroxetine	12–36	Percentage of headache days	Reduction in number of headache days per month was reported in 38% of patients. No significant analysis was reported.

[37]				No	Painful diabetic neuropathy	101	Paroxetine, citalopram	24	Pain intensity scale (0–4)	In patients who took one of the two SSRIs, 43,5% noticed no effect on the pain control, 50% felt better, and 6,5% felt worse.

[54]	No	No	No	No	Chronic tension type headache	31	Paroxetine	36	Headache index, taking in consideration days per month with headache and analgesic consumption	In patients who did not respond to amitriptyline, paroxetine failed to reduce chronic tension type headache or analgesic consumption (only 15% showed more than 50% reduction in headache index). In patients who did no respond to placebo, paroxetine produced modest reductions in headache index (39% of patients had 50% or higher reduction in headache index).

[56]	Double	Yes	No	No	Chronic tension type headache	40	Fluvoxamine	12	Pain severity and analgesic consumption	Pain intensity at baseline 2.42 and pain intensity after fluvoxamine 0.96 (*P* < 0.01). There was also a reduction in analgesic consumption (*P* < 0.05)^#^.

[61]	No	Yes	No	No	Chronic lower back pain	85	Escitalopram	13	Physician rated overall pain relief	There was no significant difference between escitalopram and duloxetine group. Significant difference was found when comparing baseline to the end of trial on escitalopram (mean change −2.30 ± 0.33) and duloxetine group (−2.45 ± 0.30).

[46]	Blind-rater	Yes	No	No	Musculoskeletal pain	40	Fluoxetine	6	Pain intensity and pain relief	Moderate or good pain relief was reported by 14 of the 17 patients (82%) in the amitriptyline group and by 14 of the 18 (77%) in the fluoxetine group. Both treatments reduced pain intensity. There was no significant difference between groups.

[58]	No	No	No	No	Central poststroke pain	31	Fluvoxamine	2–4	Pain intensity	Pain intensity at baseline 7.7 ± 2.2 was significantly reduced after fluvoxamine treatment (pain intensity 6.0 ± 3.4, *P* < 0.01).

[33]	Double		Yes	Yes	Diabetic neuropathy	17	Citalopram	6	Self-rated neuropathy symptoms	Citalopram significantly relieved the symptoms of neuropathy as measured by both observer rating and self-rating compared to placebo.

[18]	Single	Yes	No	No	Chronic tension type headache	37	Fluoxetine	12	Pain intensity, analgesic consumption, and survey short form 36 (SF36)	Baseline pain 6.6 ± 1.4; pain after fluoxetine 4.2 ± 2.9 (*P* = 0.001). The number of analgesic tablets taken per week reduced from 20 to 9 (*P* < 0.001).

[47]	No	No	No	No	Chronic prostatitis	42	Fluoxetine	12	Chronic prostatitis symptom index (CPSI)	Significant decrease in total CPSI score (28.55 to 9.29) and CPSI pain subscore (14.69 to 5.19) was observed 12 weeks after the baseline assessment (*P* < 0.05).

^#^Standard deviation not reported in the original article.

**Table 2:** Synopsis of the observed effect of the SSRI as treatment for chronic pain conditions.

SSRI	Significant reduction in pain	No significant effect on pain	Inconclusive results
Zimelidine	Different chronic pain syndromes [21]		Different chronic pain syndromes [22]

Sertraline	Noncardiac chest pain [26, 27] Chronic pelvic pain [28]	Chronic pelvic pain [29]	

Citalopram	Somatoform pain disorder [32] Diabetic neuropathy [33]	Chronic tension type headache [34] Chronic pelvic pain [35] Fibromyalgia [36]	Painful diabetic neuropathy [37] Fibromyalgia [38]

Fluoxetine	Fibromyalgia [41, 42] Migraine without aura [43] Persistent somatoform pain disorder [44] Painful diabetic neuropathy [45] Musculoskeletal pain [46] Chronic pelvic pain syndrome [47] Chronic daily headache [19] Chronic tension type headache [18]		Migraine [19]

Paroxetine	Noncardiac chest pain [49] Chronic headache [50] Fibromyalgia [52] Diabetic neuropathy [53]	Chronic low back pain [16] Chronic low back pain [17]	Chronic tension type headache [54] Painful diabetic neuropathy [37] Chronic headache [51]

Fluvoxamine	Chronic tension type headache [56] Prostatodynia [57] Central poststroke pain [58]		

Escitalopram	Chronic lower back pain [61] Multisomatoform disorder [62] Painful polyneuropathy [63]		

**Table 3:** Assessment of the risk of bias of the included studies.

Reference	Random sequence generation	Allocation concealment	Blinding of participants and personnel	Blinding of primary outcome assessment	Incomplete outcome data	Selective reporting
[38]	−	?	−	−	−	−
[32]	−	?	−	−	−	−
[41]	?	−	−	−	+	−
[16]	−	−	−	−	−	−
[34]	?	−	−	−	−	−
[35]	?	+	+	+	−	−
[43]	?	−	−	−	−	−
[17]	−	−	−	−	−	−
[49]	?	−	−	−	−	−
[29]	?	?	−	−	+	−
[51]	+	+	+	+	?	−
[22]	?	?	−	−	−	−
[37]	+	?	+	+	−	−
[42]	−	−	−	−	+	−
[54]	+	+	+	+	−	−
[21]	?	?	−	−	?	−
[26]	−	+	+	?	−	−
[50]	?	−	−	?	?	−
[28]	?	?	+	+	?	−
[44]	?	?	−	−	?	−
[56]	+	−	−	−	?	−
[45]	?	−	−	−	?	−
[61]	?	?	+	+	−	−
[62]	−	−	−	−	−	−
[36]	?	−	−	−	?	−
[63]	−	−	−	−	−	−
[52]	−	−	+	+	−	−
[19]	−	−	−	−	−	−
[46]	?	?	+	−	?	−
[58]	+	+	+	+	−	−
[53]	?	−	−	−	?	−
[33]	?	−	−	−	+	−
[57]	−	−	−	−	?	−
[27]	−	−	−	−	?	−
[18]	?	−	−	−	−	−
[47]	+	+	+	+	−	−

−: low risk, +: high risk, and ?: unknown risk.

## Additional Points

This review aims to summarise what is known, regarding the effectiveness of SSRI, as a treatment for chronic pain conditions in adults. A total of 36 studies were included in this review. Because the trials included in this review are quite heterogeneous, only qualitative analyses were performed. SSRI seems to have an effect on most of chronic pain conditions; however further clinical trials with good methodology leading to low risk of bias are needed in order to conclude once and for all the effect of this drug class as treatment for chronic pain conditions.
