# Supplementary material for: Treating Chronic Pain with SSRIs: What Do We Know?
Source: Pain Res Manag. 2016 Jul 3;2016:2020915. doi: 10.1155/2016/2020915 (PMC4947493; doi:10.1155/2016/2020915)
Supplement: Supplementary file 1 — Supplementary Table 1 presents the detailed search terms and results from the literature search. Filter restriction used was: clinical trials and English as language. [file 2020915.f1.docx]

**Supplementary Table 1.**

| **Search terms** | **Total results (initial search)** | **Total results (12 months after first search)** | **Studies included** |
| --- | --- | --- | --- |
| (("pain"[MeSH Terms] OR "pain"[All Fields]) AND ("citalopram"[MeSH Terms] OR "citalopram"[All Fields])) AND (Clinical Trial[ptyp] AND English[lang]) | 51 | 53 | [[32-34](#_ENREF_32),[36-38](#_ENREF_36)] |
| (("pain"[MeSH Terms] OR "pain"[All Fields]) AND ("fluvoxamine"[MeSH Terms] OR "fluvoxamine"[All Fields])) AND (Clinical Trial[ptyp] AND English[lang]) | 10 | 10 | [[56-58](#_ENREF_56)] |
| (("pain"[MeSH Terms] OR "pain"[All Fields]) AND ("zimelidine"[MeSH Terms] OR " zimelidine"[All Fields])) AND (Clinical Trial[ptyp] AND English[lang]) | 6 | 6 | [[21](#_ENREF_21)] |
| (("pain"[MeSH Terms] OR "pain"[All Fields]) AND ("escitalopram"[MeSH Terms] OR "escitalopram"[All Fields] OR "escitalopram"[All Fields])) AND (Clinical Trial[ptyp] AND English[lang]) | 23 | 24 | [[61-63](#_ENREF_61)] |
| (("pain"[MeSH Terms] OR "pain"[All Fields]) AND ("paroxetine"[MeSH Terms] OR "paroxetine"[All Fields])) AND (Clinical Trial[ptyp] AND English[lang]) | 54 | 54 | [[16](#_ENREF_16),[17](#_ENREF_17),[37](#_ENREF_37),[49-52](#_ENREF_49),[54](#_ENREF_54)] |
| (("pain"[MeSH Terms] OR "pain"[All Fields]) AND ("sertraline"[MeSH Terms] OR "sertraline"[All Fields])) AND (Clinical Trial[ptyp] AND English[lang]) | 26 | 27 | [[26-29](#_ENREF_26)] |
| (("pain"[MeSH Terms] OR "pain"[All Fields]) AND ("fluoxetine"[MeSH Terms] OR "fluoxetine"[All Fields])) AND (Clinical Trial[ptyp] AND English[lang]) | 53 | 54 | [[18](#_ENREF_18),[19](#_ENREF_19),[41-47](#_ENREF_41)] |
| (("pain"[MeSH Terms] OR "pain"[All Fields]) AND ("serotonin uptake inhibitors"[Pharmacological Action] OR "serotonin uptake inhibitors"[MeSH Terms] OR ("serotonin"[All Fields] AND "uptake"[All Fields] AND "inhibitors"[All Fields]) OR "serotonin uptake inhibitors"[All Fields] OR "ssri"[All Fields])) AND (Clinical Trial[ptyp] AND English[lang]) | 402 | 418 | [[21](#_ENREF_21),[22](#_ENREF_22),[32-34](#_ENREF_32),[36-38](#_ENREF_36),[50](#_ENREF_50),[51](#_ENREF_51),[57](#_ENREF_57),[58](#_ENREF_58),[70](#_ENREF_70)] [[16-19](#_ENREF_16),[26-29](#_ENREF_26),[37](#_ENREF_37),[41-47](#_ENREF_41),[52](#_ENREF_52),[54](#_ENREF_54)] |
